# Intermolecular atom–atom bonds in crystals – a chemical perspective^1^

**DOI:** 10.1107/S205225251500189X

**Published:** 2015-02-26

**Authors:** Tejender S. Thakur, Ritesh Dubey, Gautam R. Desiraju

**Affiliations:** a Molecular and Structural Biology Division, CSIR-Central Drug Research Institute, Lucknow 226 031, India; b Solid State and Structural Chemistry Unit, Indian Institute of Science, Bangalore 560 012, India

**Keywords:** intermolecular atom–atom bonds, crystal binding

## Abstract

Short atom–atom distances between molecules are almost always indicative of specific intermolecular bonding. These distances may be used to assess the significance of *all* hydrogen bonds, including the C–H⋯O and even weaker C–H⋯F varieties.

Atom–atom contact distances are widely used to assess the significance of intermolecular interactions in experimental crystal engineering. In this context, crystal structures are explained on the basis of kinetically derived structural units, that we call supramolecular synthons (Desiraju, 1995 ▶; Dunitz & Gavezzotti, 2012 ▶). Evaluating the stability of molecular crystals on the basis of contact distances between *individual* atom–atom pairs is, more often than not, adequate enough although rigorously speaking, the total stabilization, which includes contributions from both Coulombic and van der Waals terms, is more properly represented as a summation over *all* intermolecular contacts between atom–atom pairs present within a certain distance range.

Dunitz maintains that individual atom–atom pair interactions in crystals are ‘seldom structure determining’. Among crystal engineers, the most important ‘structure determining’ interactions are the strong hydrogen bonds (N–H⋯O, O–H⋯O) and their importance has been appreciated for more than a half century (Robertson, 1953 ▶). Dunitz, however, goes on to say that these are ‘the main exceptions’ to his thesis and oversimplifies the discussion even as he states that there are countless examples of structures that contain intermolecular hydrogen bonds. He further clouds the matter and invokes implicitly a Pauling-type definition of the hydrogen bond that excludes the weaker variety. There is now a very well substantiated body of research which affirms that the Pauling strong hydrogen bond is just one extreme in the hydrogen bond spectrum (Desiraju, 2002 ▶; Arunan *et al.*, 2011 ▶). C–H⋯O hydrogen bonds are a reality and cannot be neglected. There are by now too many definitive examples in the chemical and biological worlds (Desiraju & Steiner, 1999 ▶). Indeed, it seems superfluous for us to reiterate this in an essay that is written in 2015. Of course, in weak hydrogen bonds such as C–H⋯O, the contribution from the Coulombic part is moderate in comparison to the other energy components, such as polarization, induction and correlation. The role played by the more distant atoms (beyond *X*–H⋯*Y*–*Z*) in stabilizing the molecular complex cannot also be neglected, for example, in the case of the blue-shifted hydrogen bonds (Hobza & Havlas, 2000 ▶). C–H⋯O hydrogen bonds holding together molecular complexes have been proven to be stabilizing and directional both in the gas phase and in crystals (Dey *et al.*, 2014 ▶). There are many examples of C–H⋯O hydrogen bonds between molecular pairs in the gas phase which do not involve other stronger accompanying hydrogen bonds to bring them to short distances. Most importantly, we should note that the interaction geometries of these C–H⋯O hydrogen bonds in crystals correlate well with the strength of the molecular pairs bound through these interactions (Desiraju & Murty, 1987 ▶). The characteristic features of a true hydrogen bond, namely elongation of the *X*–H bond length, a red shift in the C–H stretching frequency, and deshielding of the H atom in the NMR spectrum are shown by most C–H⋯O interactions and hence these are correctly taken as C–H⋯O hydrogen *bonds*. To summarize, both strong and weak hydrogen bonds can be structure determining. Still, short (C) H⋯*X* contact distances found in crystals should always be interpreted with care because *interatomic* distances are being used to represent an *intermolecular* interaction (Steiner & Desiraju, 1998 ▶).

Intermolecular interactions such as C–H⋯O and C–H⋯F–C are weaker in the hydrogen bond hierarchy and are of secondary importance in directing the supramolecular assembly. However, in the absence of strong hydrogen bonds, weaker interactions can be major determinants of the overall crystal packing (Thalladi *et al.*, 1998 ▶). An illustrative example is provided by 1,2,3,5-tetrafluorobenzene (Thakur *et al.*, 2010 ▶) in which crystal packing is determined by weak C–H⋯F–C hydrogen bonds rather than by shape and size considerations. We have recently noted IR evidence for these very weak interactions (Saha *et al.*, 2015 ▶). As far as the strength of weak hydrogen bonds is concerned, the overall crystal stability depends not only on the strength of individual intermolecular interactions but also on their number; stronger interactions dominate over weaker ones in terms of stability but weaker interactions dominate in terms of total number. This becomes especially critical in crystal structures of the large biological macromolecules.

Dunitz refers to the atoms in molecules (AIM) approach (Bader, 1990 ▶) and while there are opposite points of view, a beginning can be made if one states that when a bond critical point (BCP) is absent, there is no interaction. In most cases of moderate to strong interaction, the presence of a BCP would be indicative of a stabilizing interaction. We would emphasize that the topological properties of electron density at a BCP (ρ_bcp_, ∇^2^ρ_bcp_) are characteristic features of stabilizing intermolecular interactions. The Koch–Popelier rules (Koch & Popelier, 1995 ▶) have been used widely as a guide to establish the presence of a stabilizing interaction and are satisfied by weaker interactions such as C–H⋯O, Cl⋯Cl and C–H⋯F–C.

Most short contact distances in crystals arise from stabilizing interactions that originate from attraction. Merely because some short contacts are repulsive, while a smaller number may actually be destabilizing, should not lead one to dismiss all short contact distances as not being of any significance in determining crystal packing. This is akin to throwing out the baby with the bathwater. Any generalization that *all* short C–H⋯C, C–H⋯O, C–H⋯F, C–H⋯Cl or C–Cl⋯Cl interactions found in crystals are repulsive or destabilizing, or that they arise purely as a result of accompanying stronger interactions or vague close packing considerations is certainly not justified. Unusually short interactions of any type always require an explanation, and croconate salts are unusual. The short intermolecular distances between anions found in the crystal structures of croconate salts are clearly forced contacts arising from other strongly attractive interactions (Dunitz *et al.*, 2014 ▶). It is suggestive that these forced contacts occur in ionic crystals where there is all likelihood of obtaining other strong electrostatic interactions that force these contacts.

Dunitz poses the question as to whether ‘... the observation of short distances between pairs of atoms on the peripheries of different molecules in crystals be regarded as evidence of specific intermolecular bonding between the atoms concerned? And if the answer is not yes but no or perhaps or sometimes: how are we to distinguish the bonding atom–atom interaction from the energetically neutral or *anti*-bonding type?’ *We believe that in most cases the answer is ‘yes’, in a few ‘perhaps’ and in the rarest of cases ‘no’.* In the end, one might suggest, somewhat rhetorically, that if Dunitz’s contentions were to be generally true, there should be a much greater tendency in polymorphic systems to obtain the thermodynamic polymorph and yet, we all know that these polymorphs are often not obtained so easily.
